# A large asymptomatic portal vein aneurysm in an old man

**DOI:** 10.1002/ccr3.3365

**Published:** 2020-11-28

**Authors:** Andrea Schilardi, Alessandro Ciavarella, Mariangela Carbone, Gianfranco Antonica, Elsa Berardi, Carlo Sabbà

**Affiliations:** ^1^ Clinica Medica ‘Cesare Frugoni’ Interdisciplinary Department of Medicine University of Bari Bari Italy; ^2^ Section of Diagnostic Imaging Interdisciplinary Department of Medicine University of Bari Bari Italy

**Keywords:** aneurysm, dilatation, portal system, portal vein

## Abstract

Ultrasound (US) is a useful tool in diagnosis and follow‐up of portal vein aneurysms (PVA). In the absence of international surgical guidelines on PVAs, US can be effectively used in follow‐up of asymptomatic patients not suitable for surgery.

## Graphical abstract

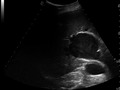

## 1 INTRODUCTION

Portal vein aneurysms (PVAs) are focal saccular or fusiform dilatations of the portal venous system. In 1976, Doust and Pearce reported a maximum diameter of the portal vein of 15 mm in healthy subjects and 19 mm in cirrhotic patients. Therefore, the portal vein diameters exceeding such measures need to be considered as pathological.^1^ Patients with PVA are frequently asymptomatic, while 50% of the patients presents with nonspecific abdominal pain.^2^ On the other hand, PVAs may also have serious clinical manifestations owing to portal hypertension or the compression of adjacent organs (eg, abdominal swelling, cholestasis, and jaundice).^3^ The most frequent PVA complication is thrombosis, reaching 20% of the cases,^2^ while spontaneous rupture is very uncommon, having been reported in only two cases.^4^, ^5^ In addition to these cases, the very first reported PVA was discovered by necropsy in 1956 in a patient dead for PVA rupture in the biliary system.^6^ Although their incidence is progressively rising for the increased number of abdominal imaging procedures,^7^ PVAs are rare findings, with an estimated incidence of 0.06%.^2^ For this reason, etiology, natural history, and treatment choices of PVAs remain still relatively unclear and no international guidelines are available on surgical indications. Herein, we report the case of an asymptomatic extrahepatic PVA, which was discovered in an 86‐year‐old man during a work‐up for dyspnea and submitted to periodical ultrasound (US) follow‐up.

## 2 CASE PRESENTATION

**FIGURE 1:**
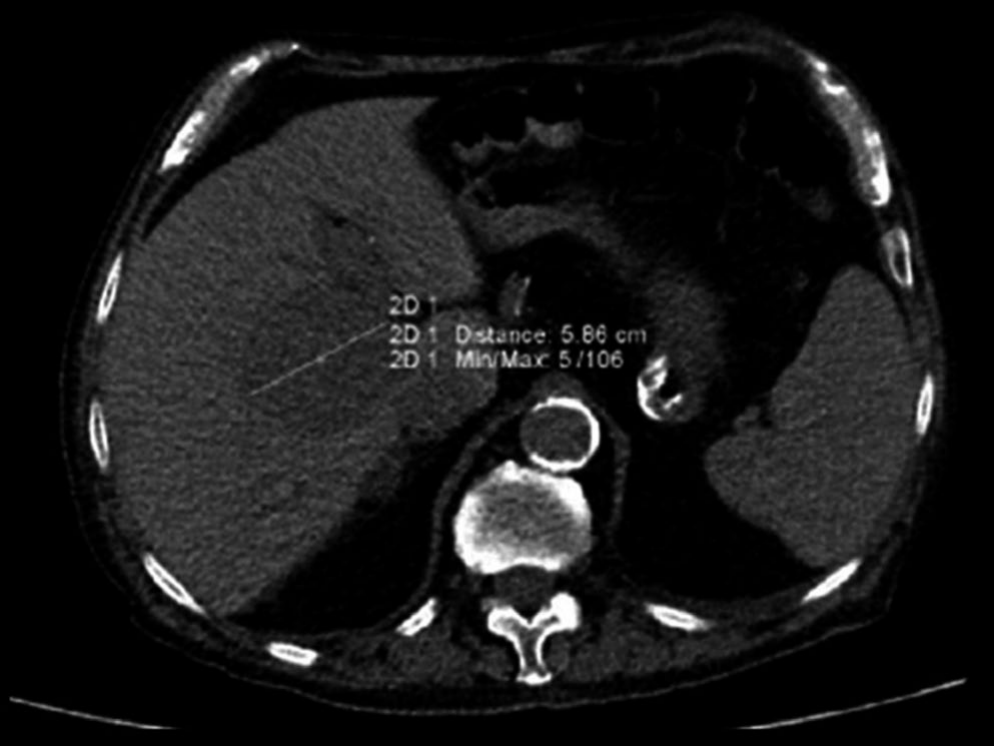
Axial CT scan without contrast agent showing an hypodense lesion of the hepatic hilum

An 86‐year‐old Caucasian man was admitted to the emergency room (ER) for dyspnea. The patient's past medical history included ischemic heart disease with congestive heart failure, permanent atrial fibrillation on anticoagulant prophylaxis with warfarin, chronic obstructive pulmonary disease (COPD), and chronic kidney disease (stage IV K‐DOQI) and was unremarkable for diseases affecting the digestive system. The patient also denied any surgical history, trauma, and malignancy. Routine laboratory tests including complete blood count, liver function, and coagulation tests were within the normal range. In the suspicion of acute exacerbation of COPD, the ER work‐up included a chest x‐ray that showed right basal parenchymal thickening and pleural effusion. Hence, during the observation period in ER, because of the worsening of respiratory failure, a computed tomography (CT) scan (without contrast medium) was required. The latter showed lung architectural distortion, reticular opacities in the immediate subpleural lung, honeycombing, and traction bronchiectasis. The thoracic radiological findings were consistent with the diagnosis of usual interstitial pneumonia (UIP). Furthermore, the scans passing through the upper abdominal quadrants showed, as an incidental finding, a space‐occupying lesion in the hepatic hilum (Figure 1). It was not possible to further define the nature of this lesion through the CT scan even because it was performed, in a patient affected by chronic kidney disease, without contrast medium.

**FIGURE 2:**
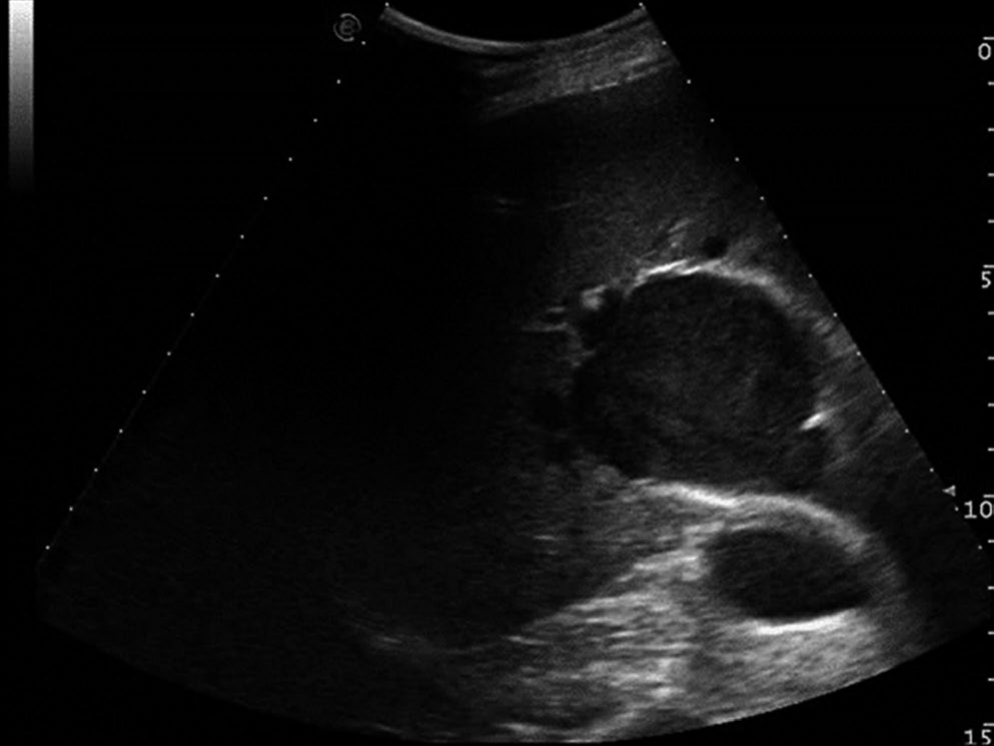
Eco‐contrast “*smoke effect*” within the portal vein aneurysm

**FIGURE 3:**
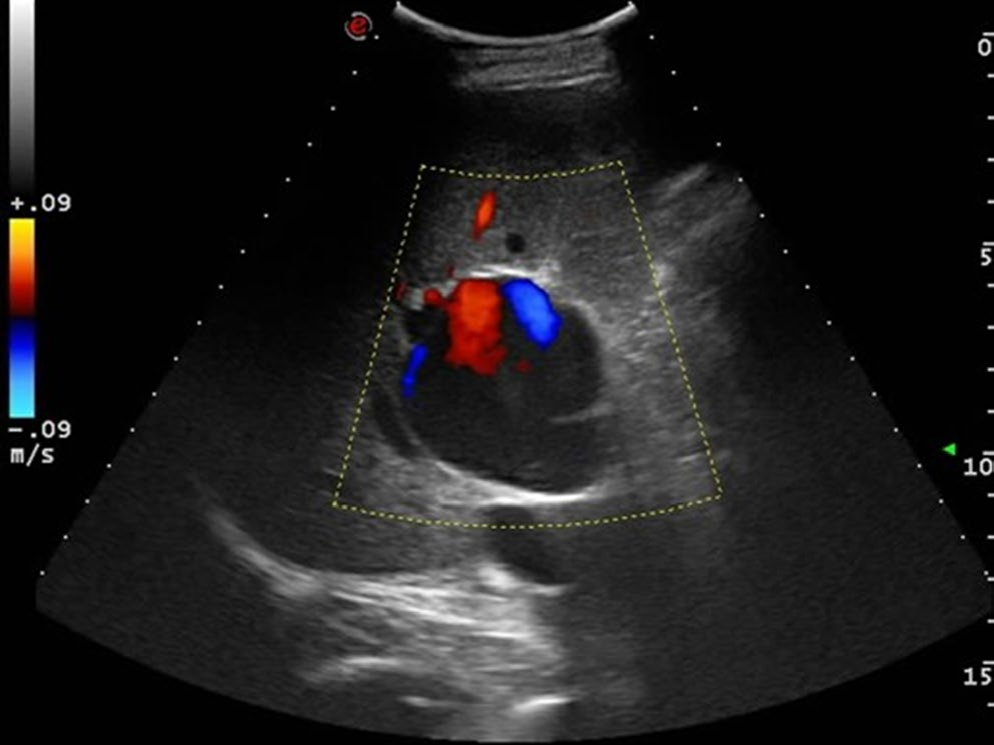
Color Doppler ultrasound showing the portal vein aneurysm with circular “*yin‐yang*” flow. Even though the color signal is detected partially, dynamic image showed “*smoke effect*” within the whole PVA, with no evidence of PVA thrombosis

An additional evaluation through abdominal US was carried out during the following stay of the patient in the medical ward. The liver hilum lesion appeared at US as a hypo‐anechoic formation, with a maximum diameter of 5.5 cm, with thickened wall, and apparently in continuity with the right branch of portal vein. The pulsed‐wave Doppler evaluation described the presence of a nonpulsatile, monophasic and slow flow inside the lesion (Figure 2), with a circular “yin‐yang” flow pattern at color flow imaging (Figure 3).

**FIGURE 4:**
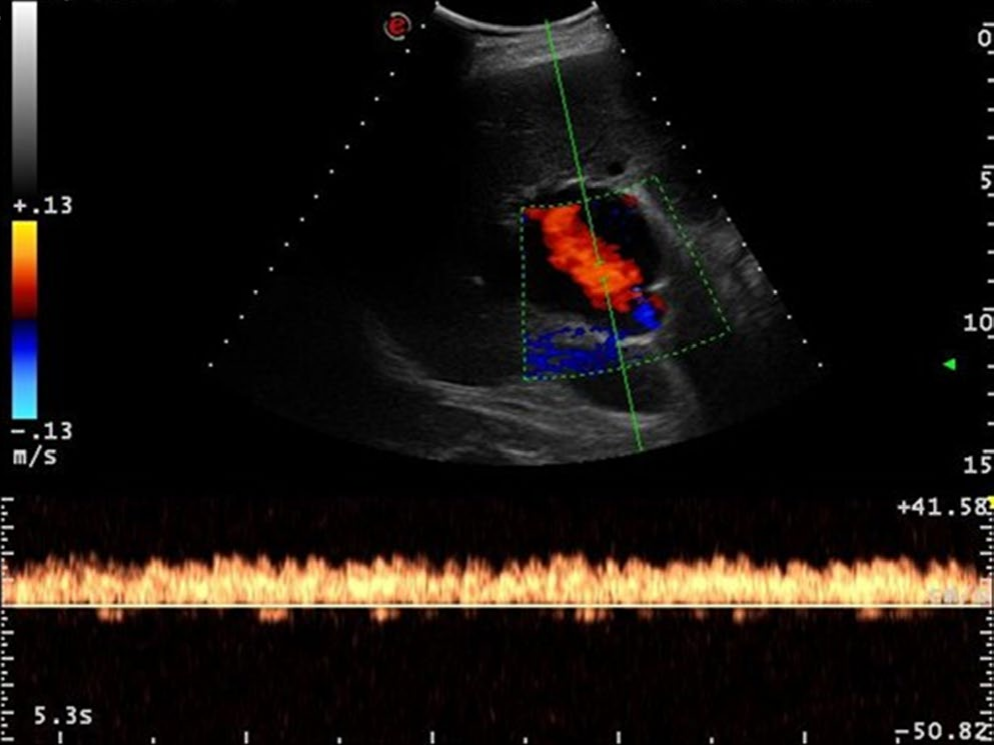
Pulsed‐wave Doppler demonstrating the presence of a monophasic, nonpulsatile venous flow pattern inside the aneurysm

Except for fatty liver disease, the US examination did not show any evidence of cirrhosis, ascites, splenomegaly, bowel distension, or any other abdominal abnormality. The aforementioned findings led to the diagnosis of portal vein aneurysm. Despite the lesion's notable dimension, no signs of portal hypertension and thrombosis were found. In fact, the spleno‐porto‐mesenteric axis was characterized by normal velocity curves throughout its whole course. Pulsed‐wave Doppler demonstrated the presence of a monophasic, nonpulsatile venous flow pattern inside the aneurysm (Figure 4). Hepatic arterial blood flow was normal (resistive index 0.63, normal values 0.55‐0.70^8^). Given the lack of clinical symptoms, the patient's age, and his comorbidities, a conservative management with periodical US evaluation every 6 months was adopted and no complications have occurred during a follow‐up period of 2 years. The patient died at the age of 88 of acute heart failure 4 months after the last follow‐up visit.

## 3 DISCUSSION

Portal vein aneurysms is a rare visceral venous aneurysm, representing 3% of the venous aneurysm in the human body,^9^ with <200 cases reported in the literature. Extrahepatic PVAs are more common than intrahepatic ones.^10^ In particular, extrahepatic PVAs are usually located at the insertion between the superior mesenteric and the splenic veins or at the hepatic hilus.^11^

The etiology of PVAs is generally considered to be either congenital or acquired. The former variant may be related to the incomplete regression of the right primitive distal vitelline vein or to the presence of other abnormalities of vitelline veins (ie, inherent weaknesses of vessels’ wall or impaired branching pattern). During the development of the portal vein taking place in the embryonic phase, such abnormalities may promote the formation of a diverticulum which can grow into a venous aneurysm.^2^ In favor of the possibility of congenital PVAs, a case of an intrauterine diagnosis in a 37‐week gestational fetus is reported in the literature.^12^ The congenital etiology is usually considered in young patients with no other comorbidities possibly related to PVAs.^13^ On the contrary, in acquired PVAs, it is possible to describe at least one underlying condition, such as traumatisms, pancreatitis, and hepatic or nonhepatic diseases.^9^ In our patient, despite his old age, the absence of any evidence of a concomitant pancreatic or liver disease was indicative of a congenital PVA. In support of this hypothesis, the aneurysm did not determine any symptom and the patient never underwent an abdominal imaging procedure before the admission to the ER. Indeed, we do not have any information about the time when the PVA developed. Also, the absence of portal hypertension in our patient may be consistent with a congenital PVA characterized by slow growth during the patient's life. However, it is not possible to exclude that the congestive heart failure may have played a role in the development on the PVA, despite the absence of portal hypertension. In 2014, Iimuro et al published the case of a PVA which grew from 3.6 to 5.1 cm during the follow‐up of 18 months. Moreover, using a computational fluid dynamics (CFD) technique, the authors conducted a study of the intra‐aneurysmatic flux, speculating that the wall shear stress against the PVA upper‐posterior part might be responsible for the aneurysm enlargement.^14^ Unlike the latter case and other cases of large aneurysms,^15^, ^16^ the dimensions of our patient's PVA remained constant over a period of 2 years. This case still represents one of the largest currently reported PVA. In addition, before the present case, only eight newly diagnosed PVAs have been described in patients aged 80 years or older and, among them, only four with no mention of cirrhosis or liver cancer.^6^, ^17^, ^18^, ^19^ According to our knowledge, no paper aiming to compare PVA in patients with and without cirrhosis is available in the English literature. A large majority of PVA are associated with cirrhosis and portal hypertension,^9^ suggesting that portal hypertension is one of the most important conditions behind PVAs development. Of note, a case of a saccular PVA undergoing an unexpected spontaneous complete regression has been reported.^20^

Given the notable dimensions of the PVA, we presumed that our patient would have had a relevant risk to develop intra‐aneurysmal thrombosis. The fact that he was taking an anticoagulant might have prevented such PVA complication. However, eighteen cases of nonthrombosed PVAs exceeding 5 cm in their largest diameter are reported in the literature, with no anticoagulation taken before their diagnosis. With regard to possible underlying factors in the risk of PVA thrombosis, in their paper Koc et al reported that four out of seven patients with thrombosed PVAs were carrying a thrombophilic defect, proposing thrombophilia screening in patients with this complication.^21^

US and CT are the methods of choice in the evaluation of a PVA.^2^ The typical findings of B‐mode US consist of a fusiform or saccular anechoic lesion, with a “smoke effect” within, which simulate a natural contrast agent determined by the slowed venous flow. Thrombosed PVAs may instead appear echogenic.^22^ The pulsed‐wave Doppler evaluation and color flow imaging help to confirm the diagnosis by demonstrating the presence of blood flow with a nonpulsatile monophasic waveform within the lesion. This evidence may help clinicians differentiating PVAs from liver benign and malignant cysts.^10^ Also, US allows to evaluate the patency of the portal vein and to study the blood flow into the aneurysm. In addition, US represents a valid noninvasive method in monitoring the PVA expansion, avoiding irradiation from CT scans. Nonetheless, CT is capable of define the accurate location of the PVA, its dimensions, and relations with adjacent organs, in order to plan a surgical intervention. In addition, the usage of magnetic resonance (MRI) has been reported only in few cases.^23^, ^24^

The clinical and therapeutic management of PVAs remains controversial. In 2012, Ma et al proposed an algorithm to help clinicians make diagnosis and therapeutic decisions in this setting.^18^ The algorithm provides an initial assessment by color Doppler US, while CT scan is reserved for indeterminate and symptomatic PVAs. According to the authors, asymptomatic patients may undergo follow‐up imaging by US. In symptomatic patients (ie, those with abdominal pain, symptoms of compression on abdominal organs, or with expanding PVAs), surgical intervention is required. Aneurysmorrhaphy or aneurysmectomy are the possible therapeutic options,^25^ while shunt surgery and thrombectomy should be reserved to patients with portal hypertension and PVA thrombosis, respectively.^26^ Moreno et al suggested an observation approach for asymptomatic aneurysms with a diameter below 3 cm.^27^ Our patient's PVA was larger than the cut‐off dimension proposed by the authors, but still asymptomatic. Hence, in view of patient's clinical condition, overlook surgery seemed the most reasonable choice.

This case highlights the importance of ultrasonography in the diagnosis and follow‐up of PVAs. Furthermore, this case represents an additional report of efficacious clinical management of a large PVA. In the absence of complications, a conservative approach to PVAs may guarantee a good clinical outcome, especially in elderly patients.

## CONFLICT OF INTEREST

The authors have no conflicts of interest to disclose.

## AUTHOR CONTRIBUTIONS

Andrea Schilardi and Gianfranco Antonica: performed the ultrasound examination. Mariangela Carbone: performed the CT scan. AS, GA, Alessandro Ciavarella, Elsa Berardi, and Carlo Sabbà: participated at the case management. AC, AS, and MC: wrote the manuscript. GA, EB, and CS: read and approved the manuscript.

## ETHICAL APPROVAL

All procedures were under the ethical standards of the local research committee.
